# National Institute of Standards and Technology Synchrotron Radiation Facilities for Materials Science

**DOI:** 10.6028/jres.106.061

**Published:** 2001-12-01

**Authors:** Gabrielle G. Long, Andrew J. Allen, David R. Black, Harold E. Burdette, Daniel A. Fischer, Richard D. Spal, Joseph C. Woicik

**Affiliations:** National Institute of Standards and Technology, Gaithersburg, MD 20899-8523

**Keywords:** ceramics, materials science, metallurgy, polymers, semiconductors, synchrotron radiation, x-ray instrumentation

## Abstract

Synchrotron Radiation Facilities, supported by the Materials Science and Engineering Laboratory of the National Institute of Standards and Technology, include beam stations at the National Synchrotron Light Source at Brookhaven National Laboratory and at the Advanced Photon Source at Argonne National Laboratory. The emphasis is on materials characterization at the microstructural and at the atomic and molecular levels, where NIST scientists, and researchers from industry, universities and government laboratories perform state-of-the-art x-ray measurements on a broad range of materials.

## 1. Introduction

Synchrotron radiation sources provide intense beams of x rays for leading-edge research in a broad array of scientific disciplines. The National Institute of Standards and Technology (NIST) Materials Science and Engineering Laboratory (MSEL) program to characterize materials by means of this powerful probe began in the early 1980s, with the design, construction and commissioning of a monochromatic x-ray topography [1] station (X23A3) at the National Synchrotron Light Source (NSLS) at Brookhaven National Laboratory. The topography station, which was home to the only dedicated monochromatic topography instrument of its kind in the country, also included experimental facilities for hard x-ray microscopy [2] and parallel-beam x-ray microradiography. By 1989, an ultra-small-angle x-ray scattering (USAXS) instrument [3] had been added to X23A3, specifically for the purpose of enabling anomalous ultra-small-angle x-ray scattering. It was later used for a wide variety of USAXS measurements on ceramics [4], metals [5], polymers [6] and biomaterials [7].

Today, the NIST/MSEL synchrotron radiation program includes the utilization and operation of three additional beam stations at the NSLS. X23A2 serves a large x-ray absorption fine structure (XAFS), diffraction anomalous fine structure (DAFS), and x-ray spectroscopy community. It provides stable scanning of monochromatic x rays in the energy range from 4.9 keV to over 30 keV. X24A, which is a shared beam station, provides incident x rays in the 1.7 keV to 6 keV energy range, and is used by NIST researchers for standing-wave x-ray measurements. Finally, the U7A station, which is also a joint effort, utilizes x-ray photoemission spectroscopy (XPS) and near-edge x-ray absorption fine structure (NEXAFS) to study the structure and chemical nature of diverse materials from a surface and bulk perspective either in vacuum or under atmospheric reaction conditions. Taken together, these three NIST/MSEL beam lines at the NSLS provide continuously tunable x rays from 0.18 keV to over 30 keV, which covers spectroscopically nearly the entire periodic table.

Looking toward the future, NIST/MSEL became a partner in 1995 with the University of Illinois at Urbana/Champaign, Oak Ridge National Laboratory, and UOP LLC, in a collaboration designated “UNICAT,” to design and construct x-ray instrumentation for sector 33 at the Advanced Photon Source (APS) at Argonne National Laboratory. The APS is presently one of the premier synchrotron x-ray sources in the world. In 1998, a new NIST USAXS facility was installed and commissioned on the sector 33 undulator beam line. In 2001, the next generation monochromatic topography and XAFS began commissioning on the sector 33 bending magnet beam line. The emphasis throughout is on state-of-the-art x-ray measurements on advanced materials. Today, the combined portfolio of NIST facilities with our partners at the NSLS and with UNICAT at the APS offers measurement capabilities in ultra-small-angle x-ray scattering, high-resolution x-ray topography, hard and soft XAFS, and standing-wave x-ray diffraction. It also offers access to leading edge instruments for structural crystallography and time-resolved structural scattering, surface and interface scattering, diffuse scattering, and magnetic scattering.

This paper is a highly selective review, by members of the current Materials Microstructure Characterization Group, of the past two decades of research efforts of the scientists who built these facilities and currently are responsible for their operation. The emphasis is on the broad range of science performed by NIST researchers and others, enabled by facilities that were designed, constructed, commissioned and operated by NIST. Many additional contributions of colleagues and collaborators can be found in the references and acknowledgements at the end.

## 2. NIST programs at the National Synchrotron Light Source

### 2.1 Monochromatic Synchrotron X-Ray Topography

**Fig. 1:**
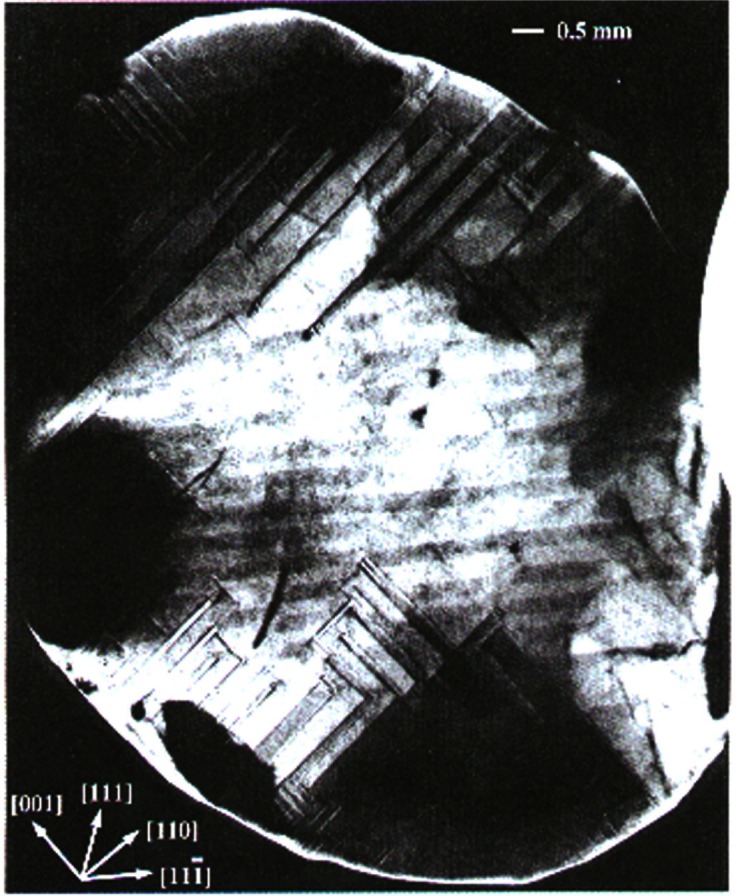
A $11\bar{1}$ transmission topograph of a Ni crystal showing 71°, 109°, and 180° magnetic domains, which intersect the surface in straight, lines along [001], [110], and $\left[11\bar{1}\right]$ respectively.

**Fig. 2:**
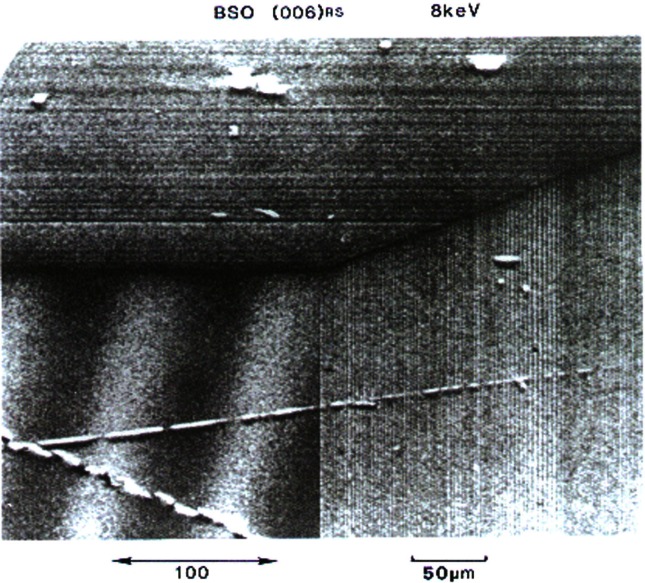
A magnified view of an (006) surface topograph from a BSO crystal showing the central (001) growth sector and the peripheral (101) and (010) growth sectors. (Also visible are scratches attributable to handling damage.)

**Fig. 3:**
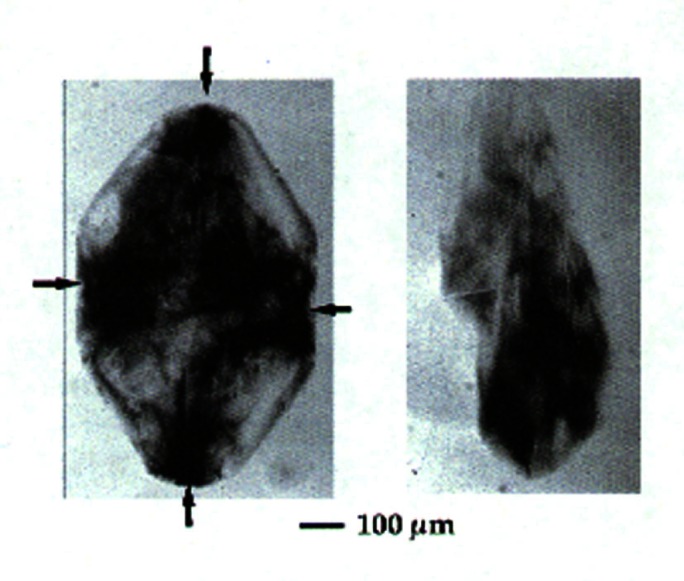
(006) X-ray diffraction topographs of a space-grown crystal on the left, and an earth-grown crystal on the right. The space crystal has symmetry about the vertical and horizontal axes consistent with growth sectors. As the Bragg angle is changed, the four dark regions indicated together migrate toward or away from the center of the crystal, consistent with a central nucleation point followed by homogeneous growth. The microstructure of the earth crystal is less well defined, consistent with a higher defect density.

From 1984, until it was closed in 2000, X23A3 was a premier monochromatic x-ray topography facility. X-ray topography is a nondestructive characterization technique for imaging, by means of x-ray diffraction, the micrometer-sized to centimeter-sized defect microstructure of crystals. The NIST Group’s early research concentrated largely on crystal growth and crystal perfection as a function of processing. The power of the monochromatic topography instrument was the result of the early realization [8] that it is possible to design and build topographic optics to record images of the entire sample in a single exposure. While the first topography camera was built in the laboratory, similar principles were used in the design of the X23A3 instrument. Early studies included the observation of magnetic domains within single-crystal grains of pure nickel. The formation of domain walls near the crystal surface was also observed (Fig. 1). Later on, the excellent resolution of the instrument—of the order of 1.7 μm—enabled detailed studies of bismuth-silicon-oxide (BSO). Three crystal slices from a single boule of BSO were examined, and the progression of microscopic strain as a function of growth was documented [9]. Remarkably, all of the features that were recorded (Fig. 2) were growth striations that could be directly related to the growth habit of the crystal boule. Recent work has included topographic examination of biological crystals grown in space [10]. One of the outstanding questions in protein crystal growth is the effect of gravity on the growth process. The crystallization of proteins can be strongly affected by gravity through sedimentation and convection. With the minimization of these processes, through growth in microgravity, will it become possible to produce better quality crystals? To answer this question, a comparison was made between ribonuclease crystals grown on the space shuttle and control crystals grown at the same time on Earth. A comparison of x-ray topographs recorded from Earth-grown and space-grown crystals is shown in Fig. 3. The space crystal has symmetry about the vertical and horizontal axes consistent with growth sectors. As the Bragg angle is changed, the four dark regions indicated migrate together toward or away from the center of the crystal, consistent with a central nucleation point followed by homogeneous growth. The microstructure of the Earth-grown crystal is less well defined, consistent with a higher defect density. The topography results were able to delineate some of the ways in which the minimization of gravity improves the quality of space-grown crystals.

### 2.2 Ultra-small-Angle X-Ray Scattering

**Fig. 4:**
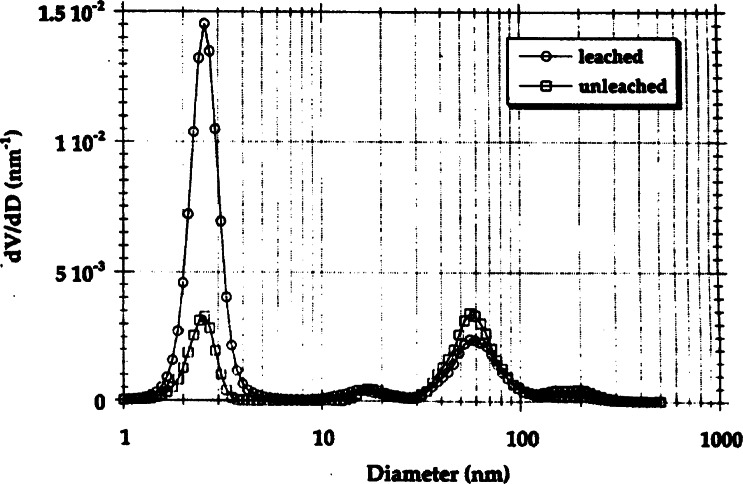
Volume distributions as a function of diameter of microporous silica, derived from USAXS and SANS data from leached and unleached material. The peak at 2.5 nm represents the population of skeletal voids in the leached material, or the alkali-silicate-rich regions in the unleached material. The peaks at ≈55 nm represent the populations of silica clusters.

The X23A3 beam line was also host to an ultra-small-angle x-ray scattering facility [3,11]. This instrument offered high angular and wavelength resolution, large sample cross sectional area, accurate energy tuning, excellent signal-to-noise ratio at ultra-small scattering vectors, and primary calibration of the absolute intensity. The scattering vector range available with this instrument served to bridge the gap between visible light scattering and standard x-ray scattering instruments. Early experiments included the quantitative determination of the microstructure of porous silica precursor bodies [12], which later led to a characterization of porosity over nearly three decades in length scale [13]. Porous materials are used in a wide variety of engineering applications, including super insulators, membranes, catalyst supports, and porous hosts for second-phase filtration. Because microstructure and performance are strongly related, the realization of different applications depends on the ability to produce porous media with specific pore sizes and pore-size distributions. The sol-gel fabrication route has been used for many polymer-based materials schemes because of the flexibility it offers to create different microstructural products. In the study of sol-gels described here, USAXS measurements on X23A3 were complemented by electron microscopy and small-angle neutron scattering measurements to quantify the microstructural features within porous silica gels. The size distributions were derived from a maximum entropy analysis of the scattering data (Fig. 4). The materials had been prepared from 20 % mass fraction colloidal silica and 80 % mass fraction potassium silicate. These precursor bodies are typically only 15 % to 20 % of theoretical density, and before densification, they are leached to remove the alkali content. Fig. 4 compares the microstructure of leached and unleached samples. The most striking feature in this derived size distribution is a narrow peak between 2 nm and 3 nm. This feature is evident in the results from both the unleached and the leached samples. It is attributed to nanometer-scale porosity in the leached samples and is attributed to similarly sized alkali-silicate-rich regions in the unleached samples. The increased scattering contrast of silica-to-void compared to silica-to-silicate accounts for the increased intensity in the scattering of the leached sample. At large diameters, there is a peak at nearly 55 nm, which represents the silica clusters. The unusual observation of skeletal voids within the solid phase, and solid clusters within the void phase in the same material is a consequence of the extraordinary variety of microstructures in these gels.

**Fig. 5:**
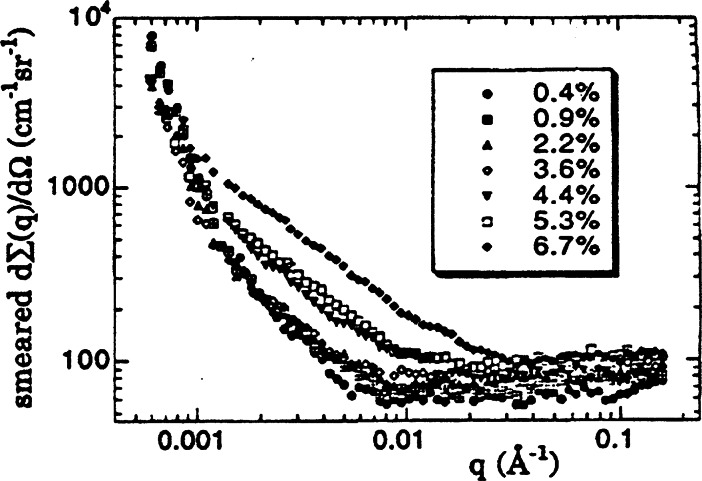
USAXS (uncorrected for slit-smearing) data from single-crystal aluminum at strains of 0.4 %, 0.9 %, 2.2 %, 3.6 %, 4.4 %, 5.3 %, and 6.7 %. The data below scattering vector ***q*** equal to 0.001 Å^−1^ is attributed to scattering from dislocation walls. The flat data between 0.01 and 0.1 are a measure of the background. Between ***q*** = 0.001 Å^−1^ and 0.01 Å^−1^ and above, the USAXS data is attributed to scattering from single dislocations. The dislocation content here is clearly seen to increase as the strain increases.

In another USAXS study, the extraordinary sensitivity of the X23A3 USAXS instrument enabled the *in situ* observation of x-ray scattering from dislocation structures during the deformation of single crystal aluminum [14]. Attempts over the years to measure small-angle scattering from dislocation structures have mostly met with limited success for various reasons. One of the earliest recognized reasons was the intrusion into the data of double-Bragg diffraction. Another problem was surface scattering from improperly prepared surfaces. Finally, unless the scattering vector is nearly perpendicular to a dislocation line, no appreciable scattering is seen. By avoiding double-Bragg diffraction conditions, meticulous sample surface preparation, and by orienting the samples for dislocation visibility, it was possible to observe: 1. correlations in the ordered fraction of dislocations, 2. the presence of dislocation dipoles, 3. the increasing dislocation content with increasing strain, and 4. the decreasing width of the interface between the dislocation walls and the surrounding, nearly-dislocation-free material. In Fig. 5, the increasing dislocation content with increasing strain can be seen directly from the slit-smeared USAXS from single-crystal aluminum at strains of 0.4 %, 0.9 %, 2.2 %, 3.6 %, 4.4 %, 5.3 %, and 6.7 %.

After 15 years of successful operation, X23A3 was closed in 2000 when the next-generation USAXS and topography instruments went into commissioning at the APS.

### 2.3 X-Ray Absorption Fine Structure

The X23A2 beam line provides a stable, scanning, highly monochromatic x-ray beam covering the energy range of 4.9 keV to over 30 keV. It was designed and built by scientists in the Electrical and Electronic Engineering Laboratory at NIST. In 1991, two of the scientists and the instrumentation became part of MSEL. Work performed on X23A2 has focused on bond distortions in the GeSi [15], the AlGaN [16], and the InGaAs [17, 18] alloy systems. A large fraction of this work addressed questions related to the strain and bond distortions in thin alloy films of these materials that had been grown epitaxially on substrates with dissimilar lattice constants.

**Fig. 6:**
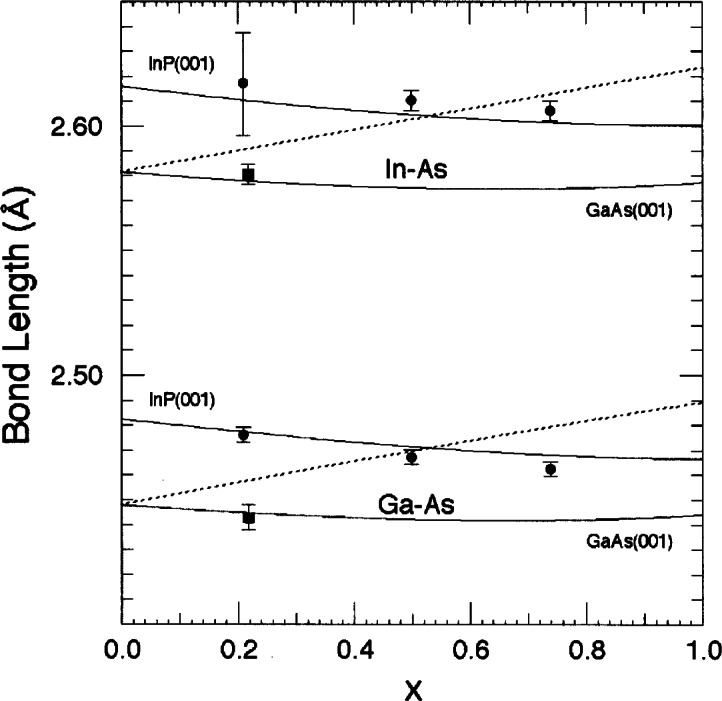
Comparison of the experimentally determined EXAFS and DAFS In-As and Ga-As bond lengths in pseudomorphic Ga_1−_*_x_* In*_x_* As alloys grown on GaAs(001) (squares) and InP(001) (circles) with the results of the theoretical, random-cluster calculations. The dashed lines are the calculated cubic (bulk) bond lengths, and the solid lines are the calculated tetragonal (strained) bond lengths. Results for the different substrates are as indicated. Note that the bulk and strained bond lengths coincide for the lattice-matched, zero strain composition.

X23A2 was used to develop the diffraction anomalous fine structure technique (DAFS) [19,20]. All of these studies required the extremely stable, broad energy scanning capabilities of the beam line. The analysis of the XAFS and DAFS data enabled the development of a microscopic model that describes the local atomic distortions in strained films that is based on the random cluster approximation and the Keating valence force field within the context of macroscopic elastic theory [21]. Fig. 6 compares the experimental bond lengths for strained Ga_1−_*_x_*In*_x_* As films grown on both GaAs (001) and InP (001) substrates with the theoretical predictions. The large departures of the strained bond lengths from the bond lengths in the cubic, unstrained alloys as a function of substrate lattice constant are noteworthy. Clearly, these distortions are found to oppose the natural bond-length distortions due to alloying.

**Fig. 7:**
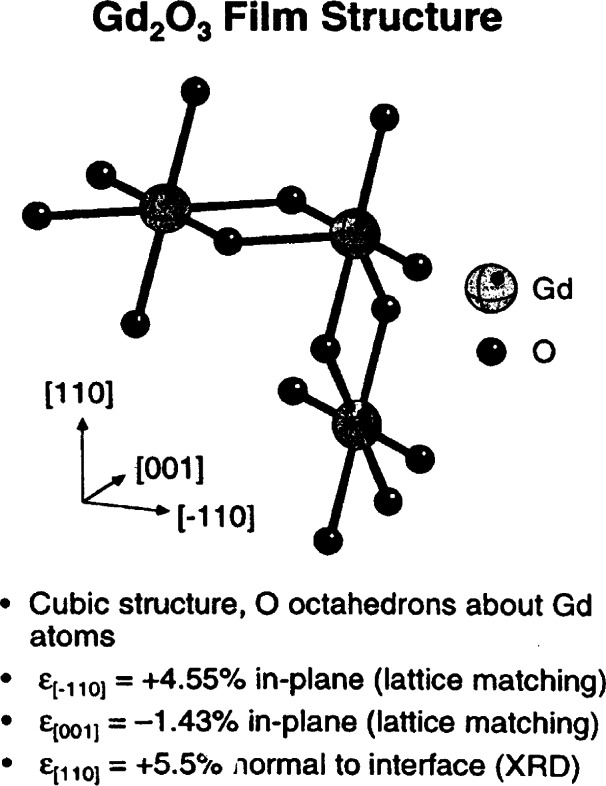
The basic building block of the Gd_2_O_3_ crystal structure. The [−110], [001], and [110] directions of Gd_2_O_3_ are shown.

More recent work using X23A2 addressed the structure of passive oxide films grown on GaAs (001) [22]. It was determined that the bond length in a 23 Å Gd_2_O_3_ film grown epitaxially on GaAs (001) was strained +2.7 % ± 0.6 % relative to the bond length in bulk Gd_2_O_3_ powder. Using a simple model for the strained film based on the phenomenology developed for strained semiconductor alloys, lattice matching of the [001] and [−110] axes of the Gd_2_O_3_ film with the [110] and [1–10] axes of the GaAs (001) surface, the measured bond length is in excellent agreement with the perpendicular lattice constant of the film as determined by x-ray diffraction (Fig. 7).

While X23A2 provides relatively hard energy x rays, X24A offers x rays in the tender energy range of 1.7 keV to 6 keV which NIST scientists have used for x-ray standing wave, x-ray fluorescence, and x-ray photoelectron spectroscopy experiments. The facility is supported by three laboratories within NIST (MSEL, the Chemical Sciences and Technology Laboratory, and the Physics Laboratory), the NSLS, and the University of Rhode Island. In the past, the x-ray standing wave technique was used to determine the atomic-scale structure of over layer atoms on single crystal surfaces [23], of interfacial atoms in buried semiconductor interfaces [24], and of reconstructed atoms at clean semiconductor surfaces [25].

**Fig. 8:**
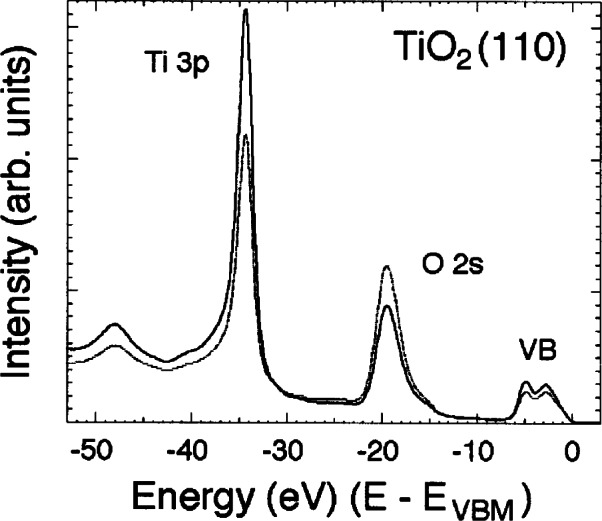
Photoelectron spectra from the rutile TiO_2_(110) surface recorded in the vicinity of the TiO_2_(200) Bragg back-reflection condition (*hv* ≈ 2700 eV). The spectra have been referenced to the valence-band maximum and extend over the kinetic-energy range of the Ti 3*p*, O 2*s*, and valence electron emission. The photon energies were chosen to maximize either the electric-field intensity on the Ti atoms (bold line) or on the O atoms (shaded line).

**Fig. 9:**
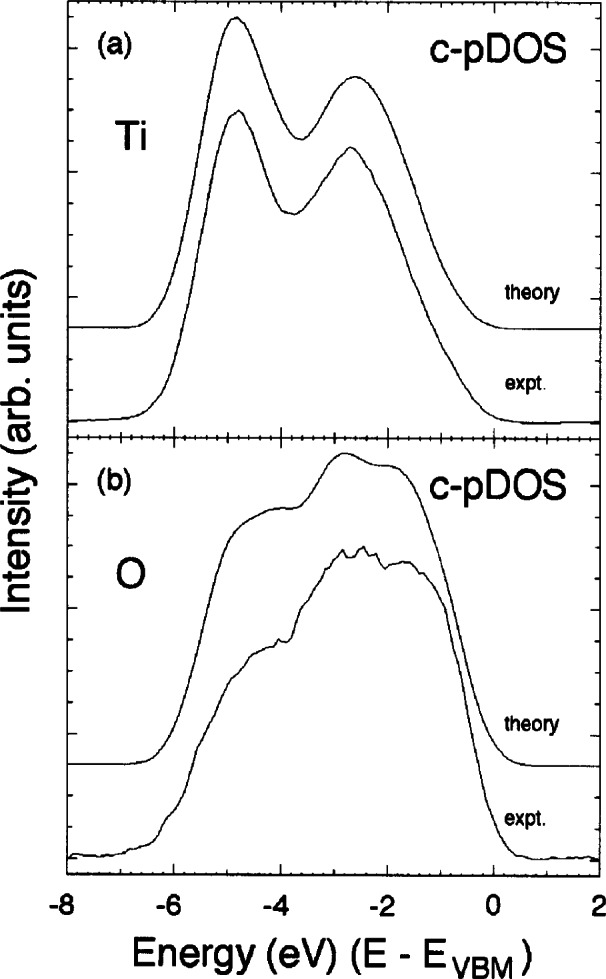
a) Comparison of the LDA calculation of the Ti partial density of states corrected for the individual Ti angular-momentum photoelectron cross sections with the experimental Ti valence-photoelectron spectrum. The spectra have been referenced to the valence-band maximum and scaled to equal peak height. b) Comparison of the LDA calculation of the O partial density of states corrected for the individual O angular-momentum photoelectron cross sections with the experimental O valence-photoelectron spectrum. The spectra have been referenced to the valence-band maximum and scaled to equal peak height.

Recently, NIST researchers combined high-resolution x-ray photoelectron spectroscopy with the site-specificity of x-ray standing-wave diffraction to develop a new technique by which the partial densities of occupied electronic states of single crystals may be obtained [26]. To illustrate the principle of the technique, Fig. 8 compares two x-ray photoelectron spectra from the rutile TiO_2_ (110) surface recorded within the energy width of the TiO_2_ (200) Bragg back-reflection condition (*hv* ≈ 2700 eV). The spectra extend over the kinetic-energy range of the Ti 3*p*, the O 2*s*, and the valence-electron emission. The photon energies were chosen to maximize either the electric-field intensity on the Ti atoms (bold line) or on the O atoms (shaded line). The data exhibit the well-known x-ray standing wave effect for both the Ti 3*p* and O 2*s* core lines, as well as for the crystal valence band. The resulting atomically resolved components of the crystal valence band are shown in Fig. 9. These data are compared to *ab initio* local density approximation calculations of the partial density of states of rutile TiO_2_ that have been corrected for the angular-momentum-dependent photoelectron matrix elements of the Ti 4*s*, 4*p*, and 3*d* orbitals, as well as the O 2*s* and 2*p* orbitals [27]. The agreement between theory and experiment is startling, revealing the chemical hybridization on each site that is directly responsible for the solid-state electronic structure.

### 2.5 Soft X-Ray Spectroscopy

The U7A soft x-ray materials characterization facility utilizes polarized soft x rays between 180 eV and 1500 eV to reveal the structure and chemical nature of diverse materials. U7A, which is operated as a partnership between NIST, The Dow Chemical Company,^1^ and Brookhaven National Laboratory, achieved fully dedicated operation in 1996. The broad array of research includes model catalyst systems [28] polymer surfaces and their interfaces [29] magnetic hard disk lubricants [30], biological materials [31] and high temperature superconductors [32]. Utilizing partial electron yield and fluorescence yield detection, surface (≈2 nm) and bulk (≈200 nm) structure and chemistry can be recorded simultaneously utilizing soft x-ray near-edge X-ray absorption fine structure (NEXAFS) [33].

**Fig. 10:**
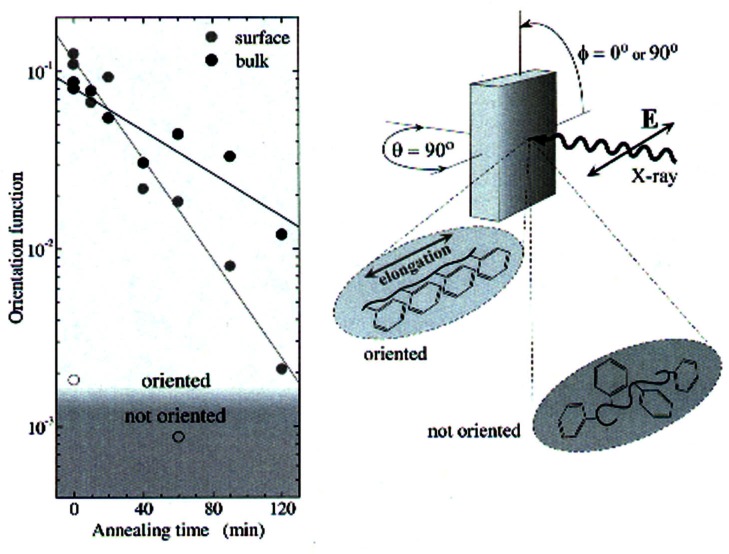
An “orientation factor,” OF, is defined as OF = (*I*_∥_ − *I*_⊥_)/(*I*_∥_+ *I*_⊥_), where *I*_∥_ and *I*_⊥_ are the 1*s*→*π*_1_* resonance NEXAFS intensities collected with the sample elongation direction parallel (*ϕ* = 0°) and perpendicular (*ϕ* = 90°) to the electric vector of the soft x-ray beam, ***E***, respectively. The experimental geometry is shown on the right; the results are shown on the left..

Recently, NIST researchers, in collaboration with scientists from the North Carolina State University, applied NEXAFS at U7A to measure directly both surface and bulk segmental relaxation throughout a uniformly deformed polystyrene slab. Using this methodology, in a single experiment, chain relaxation was found to proceed almost 50 % faster at the surface than in the bulk [34]. Fig. 10 shows the orientation function (OF) for both surface and bulk chains decays as a function of increasing annealing time at 60 °C, fitting the decay rates to exponential functions gives characteristic time constants of approximately 33 min and 50 min for the surface and the bulk, respectively. These results are in accord with recent theory [35] predicting that collective motion on chain loops extended to the sample surface is responsible for a rapid increase of chain mobility near the surface region of the film (top ≈5 nm). The finding that the polystyrene surface segmental mobility greatly outpaces the bulk is expected to be a universal property of polymeric chains, profoundly influencing the design, processing, and application of polymeric materials. In addition, the time scale that controls polymer chain relaxation has taken on a new importance as nanotechnology polymer innovation evolves from three dimensions to two.

In a recent biopolymer application with the NIST Orthopedic Wear Consortium, the U7A beam line was used to investigate the wear properties of ultra-high molecular weight polyethylene (UHMWPE). Over half a million patients receive artificial joint replacements annually, and practically all the replacements consist of a sliding pair represented by a polymer (UHMWPE) and a hard counterface (metal or ceramic). For the past 30 years UHMWPE has remained the dominant polymer in artificial joints due to its outstanding properties. Nevertheless, it is now recognized that wear of UHMWPE contributes to the eventual loosening of the implants and is the main cause for the failure of long-term implants. Hence there is an urgent need to understand the mechanism and the surface morphology leading to wear and failure of the artificial joint. Current methods of inferring or deducing orientation information for UHMWPE lack surface sensitivity and mostly rely on staining the specimens followed by SEM examination. Soft-x-ray measurements on U7A were able to indicate conclusively that on average at least 90 % ± 5 % of the polyethylene molecular chains are preferentially aligned parallel to the rubbing direction, whereas samples that have not undergone rubbing stress yield nearly isotropic or random orientation of polyethylene chains. In addition, it was deduced that on average very few of the polyethylene molecular chains are aligned perpendicular to the rubbing direction. Thus, the molecular chain orientation of rubbed UHMWPE used in artificial joints could be determined conclusively and non-destructively.

## 3. NIST Programs in the UNICAT Collaboration at the Advanced Photon Source

The UNICAT (University-National Lab-Industry Collaborative Access Team) at the Advanced Photon Source—which includes NIST, the University of Illinois at Urbana/Champaign, Oak Ridge National Laboratory, and UOP—supports a broad range of research techniques. These include: ultra-small-angle x-ray scattering, high resolution x-ray diffraction, surface x-ray diffraction, diffuse x-ray scattering, x-ray topography, XAFS, magnetic scattering, and x-ray tomography. To meet the technical demands of our diverse experimental portfolio, the UNICAT beam lines on Sector 33 exploit the brilliance of the APS source to deliver x rays with high flux and with energy resolution better than or equal to the lifetime-broadened core-hole widths for all accessible K and L shells. The insertion device beam line, 33-ID, became operational November 1, 1999, and as of November 1, 2000, has entertained independent investigators as well as collaborative access team members. The bending magnet beam line, 33-BM, went into commissioning in March 2001 and is expected to become fully operational by the end of 2001.

### 3.1 Ultra-Small-Angle X-Ray Scattering and Imaging

Ultra-small-angle x-ray scattering (USAXS) was the first of the NIST-responsible experiments to come on line [36]. It enables researchers to measure data in the usually inaccessible scattering vector, ***Q***, range (where |***Q***| = (4*π*/*λ*) sin*θ, λ* is the x-ray wavelength and 2*θ* is the scattering angle) down to nearly 1 × 10^−4^ Å^−1^. The UNICAT instrument includes several capabilities that make it among the best in the world. It covers an unprecedented |***Q***| range from 0.00012 Å^−1^ to 1.0 Å^−1^; anomalous-USAXS can be acquired to investigate separately and quantify previously inseparable microstructures; and it supports the new technique of USAXS imaging. In addition, an optional “effective-pinhole” configuration is available in which transverse crystal reflections are added, thereby removing the intrinsic slit smearing of USAXS. In the effective pinhole geometry, the resolution of the scattering vector increments can be made exceedingly fine, easily surpassing that of true pinhole instruments with a 2D detector. The USAXS instrument presently enjoys the highest demand of any instrument on UNICAT.

**Fig. 11:**
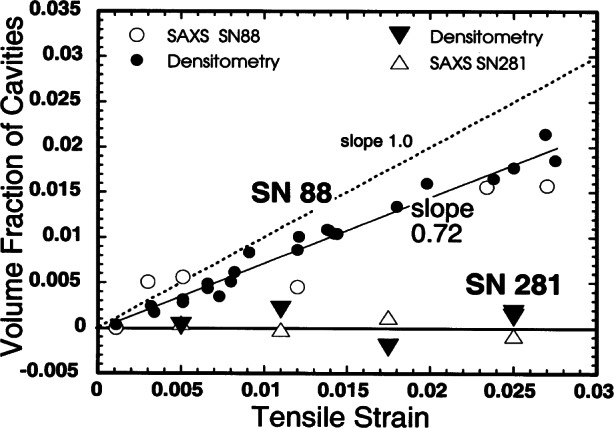
Cavity volume fractions are shown as a function of tensile strain for SN88 and SN281. The open circles are results from scattering data on SN88, and the open triangles are results from scattering data on SN281. The companion data from densitometry are given by the filled circles (SN88) and filled (SN281) triangles, respectively.

In a recent UNICAT USAXS ceramic deformation study, x-ray scattering by silicon nitride was measured as a function of tensile strain in an effort to improve the understanding of the mechanisms controlling creep. In an attempt to improve creep resistance, which ultimately limits the lifetime of ceramic Si_3_N_4_ components, rare-earth-based secondary phases have been added. In this study, two commercial silicon nitride formulations were measured, including a new one that exhibits an increase in creep resistance of more than three orders of magnitude and an increase in lifetime of over two orders of magnitude. To understand the reasons for such a dramatic improvement in creep resistance, anomalous USAXS data were recorded to measure both the secondary (additive) phase and the porous phase as a function of tensile deformation. Ordinarily, scattering from the large volume fraction (from approximately 5 % to 10 %) of secondary phase would dominate the scattering from the creep void fraction (0 % to 2.5 %), and an analysis of creep cavities could not be done. By means of anomalous scattering measurements and analysis, such quantification is rigorously possible [37]. The USAXS results indicated that in the new formulation of silicon nitride, cavitation does not occur, compared to the other grade of Si_3_N_4_. Fig. 11 shows the contribution of cavities to tensile strain in the new SN281 compared to the older SN88. Redistribution of the secondary phases via grain boundary sliding and solution-precipitation may serve as the creep mechanism.

**Fig. 12:**
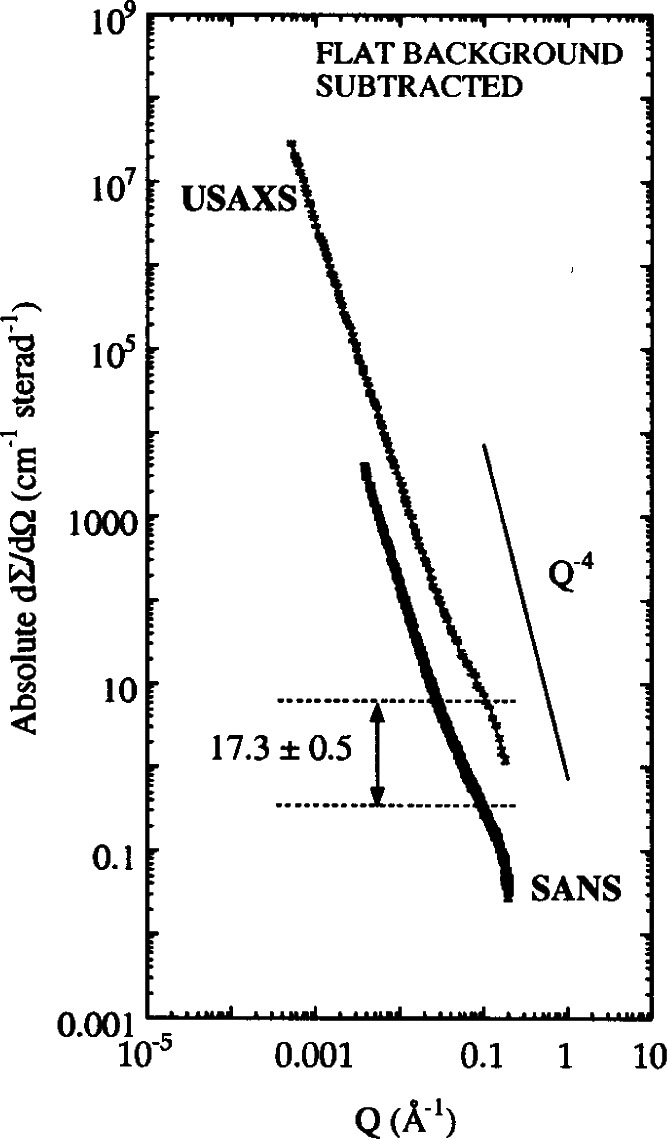
Absolute-calibrated USAXS and SANS data for a typical 28 day old OPC.

Another example of UNICAT USAXS studies involved the characterization of the nature of the amorphous calcium-silicate-hydrate (C-S-H) gel that is the main strength-giving phase in hydrating cementitious systems including concrete. Here, the ability of the USAXS instrument to provide a primary absolute calibration of the scattered intensity with respect to the incident beam (without the need for a secondary scattering standard) proves critical. Absolute-calibrated USAXS and small-angle neutron scattering (SANS) contrast variation studies were combined in a study to determine, as independently as possible, the formula and density of the C-S-H solid phase that could be compared with various previously proposed models for C-S-H [38]). Fig. 12 shows an absolute comparison of USAXS and SANS data for a 28 day old ordinary Portland cement as a function of ***Q***. The USAXS data were corrected for multiple scattering effects observed at low ***Q*** and the most affected data, for ***Q*** < 0.0005 Å^−1^, were discarded. Figure 12 shows that the fractal C-S-H gel structure observed by USAXS extends to significantly lower ***Q*** (and correspondingly higher scale-lengths) observed in the SANS range. The ratio of USAXS to SANS calibrated intensities at high ***Q*** (most sensitive to the C-S-H:H_2_O interface), when combined with other information, is found consistent with only one of the models for solid C-S-H previously proposed. Apart from solving this problem these studies also resolved a discrepancy between absolute-calibrated SANS and earlier SAXS determinations of the C-S-H:H_2_O inter-facial surface area. The earlier SAXS studies had not been absolute-calibrated but relied on an integrated scattering approach (the “scattering invariant” method) and had yielded surface-area values two to six times higher than the SANS values. In the present study these discrepancies could be traced to uncertainties in the interpolation and normalization of data required in the scattering invariant method, and the absolute-calibrated USAXS and SANS surface areas were found to be completely consistent.

**Fig. 13:**
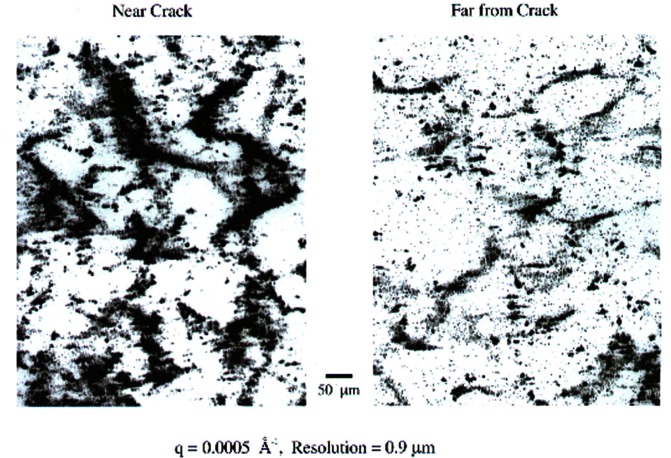
USAXS images taken with 0.9 ?m resolution of the regions near (left) and far (right) from a crack in copper metal.

The ultra-small-angle scattering curve and its transform lead directly to the volume-averaged determination of many microstructural parameters. However, USAXS cannot measure the homogeneity or lack thereof in a sample, and one must usually rely on complementary techniques for information on the distribution of microstructures and their morphology. In response to the need for this kind of microstructural information, as well as the need to quantify it, NIST researchers are developing the new technique of USAXS imaging [39]. In this application, the UNICAT USAXS instrument is used with a 2-reflection collimator before the sample and a 2-reflection analyzer after the sample. With the analyzer crystal pair set to |***Q***| equals zero, and the photodiode detector replaced by an imager, a high-resolution radiograph of the sample can be obtained. With the analyzer set to an angle within its rocking curve, a diffraction-enhanced image (which is a form of “phase contrast imaging”) is obtained. Finally, with the analyzer crystal set to an angle within the small angle scattering regime, USAXS images are obtained. The photons that produce the image have scattered from the internal density fluctuations in and on the sample (at intensities ≈10^−2^ to 10^−8^ of maximum). This is possible *only* at angles where the scattered intensity exceeds the transmitted intensity (well away from the transmitted beam). In this manner, the microstructures contributing to the scattering at that particular angle-filtered window, Δ*Q*, are imaged. By using images created at different scattering vectors, it is possible under certain conditions to view different-sized features within a single volume. By rotating the sample about a single scattering vector, stereo pairs can be produced, and 3D information is gained. Finally, even when USAXS imaging is not used for three-dimensional imaging purposes, it is a useful adjunct to USAXS in that it offers a means of identifying spurious effects such as double Bragg diffraction and surface scattering, and the opportunity to avoid them. Although metallic, ceramic, polymer and biological structures have been imaged, the emphasis of USAXS imaging research has mostly been on creep cavitation and ductile fracture in metals. In a recent study, ductile fracture of pure copper was investigated. Fig. 13 shows images taken close to and distant from a crack. The microstructures that are imaged are creep cavities that have formed at the grain boundaries. The data show that the microstructures far from the crack are similar in quality from those near the crack, but very different in quantity.

### 3.2 Looking Toward the Future at the Advanced Photon Source

At the time of this writing, two additional NIST-responsible instruments are being commissioned: x-ray topography and x-ray absorption fine structure. A third, x-ray microtomography, is still under development. Just over the horizon, NIST and UOP LLC are contemplating the possibility of joining the University of Illinois and Oak Ridge National Laboratory in the commissioning, operation and utilization of the microbeam capability of Sector 34.

## 4. Conclusions

In 1984, NIST’s synchrotron facilities were inaugurated with the commissioning of a single beam station at the NSLS for x-ray topography. Today, the combined portfolio of NIST/MSEL facilities with our partners at the NSLS and with UNICAT at the APS offers measurement capabilities in ultra-small-angle x-ray scattering, high-resolution x-ray topography, hard and soft XAFS, and standing-wave x-ray diffraction. It also offers access to leading edge instruments for structural crystallography and time-resolved structural scattering, surface and interface scattering, diffuse scattering and magnetic scattering. The NIST synchrotron facilities enable researchers to use these intense x-ray sources for the advancement of science and technology in materials, physics, chemistry, and, most recently, biology. The primary thrust is the application of leading-edge x-ray scattering and imaging to a broad range of scientific problems and the continued development of novel techniques for innovative research. MSEL welcomes use of its synchrotron-based facilities by researchers at NIST and from other National Laboratories, universities, and industry.

## Footnotes

1 Certain commercial equipment, instruments, or materials are identified in this paper to foster understanding. Such identification does not imply recommendation or endorsement by the National Institute of Standards and Technology, nor does it imply that the materials or equipment identified are necessarily the best available for the purpose.
